# Machine learning-based predictive modeling of resilience to stressors in pregnant women during COVID-19: A prospective cohort study

**DOI:** 10.1371/journal.pone.0272862

**Published:** 2022-08-11

**Authors:** Emily S. Nichols, Harini S. Pathak, Roberta Bgeginski, Michelle F. Mottola, Isabelle Giroux, Ryan J. Van Lieshout, Yalda Mohsenzadeh, Emma G. Duerden

**Affiliations:** 1 Applied Psychology, Faculty of Education, Western University, London, Ontario, Canada; 2 The Brain and Mind Institute, The University of Western Ontario, London, Ontario, Canada; 3 Department of Computer Science, The University of Western Ontario, London, Ontario, Canada; 4 R. Samuel McLaughlin Foundation—Exercise and Pregnancy Laboratory, School of Kinesiology, Faculty of Health Sciences, Children’s Health Research Institute, Western University, London, Ontario, Canada; 5 Department of Anatomy and Cell Biology, Schulich School of Medicine and Dentistry, Western University, London, Ontario, Canada; 6 School of Nutrition Sciences, Faculty of Health Sciences, University of Ottawa, Ottawa, Ontario, Canada; 7 Department of Psychiatry and Behavioural Neurosciences, McMaster University, Hamilton, Ontario, Canada; 8 Psychiatry, Schulich School of Medicine and Dentistry, University of Western Ontario, London, Ontario, Canada; Universiti Sains Malaysia, MALAYSIA

## Abstract

During the COVID-19 pandemic, pregnant women have been at high risk for psychological distress. Lifestyle factors may be modifiable elements to help reduce and promote resilience to prenatal stress. We used Machine-Learning (ML) algorithms applied to questionnaire data obtained from an international cohort of 804 pregnant women to determine whether physical activity and diet were resilience factors against prenatal stress, and whether stress levels were in turn predictive of sleep classes. A support vector machine accurately classified perceived stress levels in pregnant women based on physical activity behaviours and dietary behaviours. In turn, we classified hours of sleep based on perceived stress levels. This research adds to a developing consensus concerning physical activity and diet, and the association with prenatal stress and sleep in pregnant women. Predictive modeling using ML approaches may be used as a screening tool and to promote positive health behaviours for pregnant women.

## Introduction

During the COVID-19 pandemic, pregnant women have been exposed to significant stress, including infection-related fears, social isolation, loss of income, lack of access to medical care [1]. Previous studies that have examined prenatal stress during natural disasters or epidemics suggest that prenatal stress is a significant contributor to adverse maternal and neonatal health outcomes [2]. Recent evidence suggests that prenatal distress has become endemic in pregnant women during COVID-19 [3]. Given the current global pandemic, millions of pregnant women are at risk for adverse health outcomes with few guidelines currently in place to help women manage stress and mitigate its harmful effects.

Prenatal stress is a risk factor for pregnancy complications including low birth weight, preterm labour, hypertension, and delayed neonatal development [4, 5]. Several lifestyle factors have emerged as protective factors to promote resiliency against prenatal stress in pregnant women including physical activity and diet [6]. However, due to ongoing physical distancing regulations, pregnant women may be more likely to work from home and spend leisure time online or consuming other forms of media [7], and pregnant women who reported decreases in physical activity during the pandemic exhibited higher levels of depression [8]. Another study showed that pregnant women who performed the international recommendation of 150 min of physical activity per week [9] exhibited significantly lower anxiety and depression scores compared to women who performed less [3], suggesting that physical activity may be a key resilience factor to promote maternal well-being. However, studies examining physical activity in pregnant women during the pandemic have used limited measures and focused on change in levels [3, 8]. Because the form of physical activity that many women are engaging in has likely changed due to closures and restrictions on gatherings, more research is required to determine what forms of physical activity pregnant women are engaging in, and how this relates to stress during the pandemic.

Diet is also a modifiable lifestyle factor that predicts maternal and neonatal health [10]. Psychological distress is associated with poor nutrition in healthy adults [11]; yet, stress has also been identified as a potential risk factor for emotional eating behaviours in pregnant women during the pandemic [12]. Further, a recent report indicated that households are purchasing fewer fresh items such as meat, fruits, and vegetables [13]. Limited food access and increased unhealthy eating behaviours may have consequences for perceived stress, and a better understanding of the dietary behaviours that pregnant women are currently engaging in, and their association with stress, is needed.

These changes in behaviour are likely to have downstream effects beyond the impact on stress levels. In pregnancy, sleep loss is endemic, with up to 60% of women experiencing insomnia [14], and sleep disruptions in pregnant women have only increased during the pandemic [15]. Psychological distress related to COVID-19 has been a major cause of these disruptions [15], and associations amongst sleep characteristics (i.e., sleep time, hours of sleep) and maternal distress during the early phases of the pandemic have been reported [16]. Poor sleep is also strongly predicted by stress during pregnancy, which is associated with adverse maternal outcomes [17], indicating that increased levels of pandemic-related stress may be associated with worse sleep quality in pregnant women.

Machine learning (ML) is a useful tool in examining complex relationships between many predictors. Work in pregnancy has employed ML techniques for decades; for example, a recent article reviewed 26 studies that used ML to study the optimal mode of childbirth [18]. Other work has examined risk factors in pregnancy, including for sepsis [19], ectopic pregnancy [20], and gestational diabetes (see [21] for a review).

Based on previous work, in the present study we sought to determine whether physical activity and diet are associated with resilience against prenatal stress, and whether stress is in turn associated with sleep, during the COVID-19 pandemic. We applied a series of ML algorithms to a heterogenous dataset from an international cohort of pregnant women who completed an online survey study during May to September 2020. Our central hypothesis was that both physical activity and diet would be predictive of prenatal stress, and that in turn, stress would be predictive of sleep levels, in pregnant women, to identify modifiable lifestyle factors that are associated with reduced stress and improved sleep in pregnant women during the pandemic.

## Materials and methods

### Participants

A total of 1049 pregnant individuals enrolled in the study. Complete data were available for 804 after data cleaning procedures (see Section 2.4 for details). Full demographic information for the final sample is provided in Table 1. Recruitment and data collection were performed online via Prolific, Amazon’s Mechanical Turk, and through advertising on social media websites, and was targeted worldwide, within a single time window. Participants were mainly recruited from the United States (31.7%), the United Kingdom (20.6%), Canada (20.5%), and India (10.7%). Women ranged in age from 18–53 years (M = 30.67, SD = 5.07), and gestational age ranged from 2 to 42 weeks (M = 24.5, SD = 9.53). This study was approved by the Western University Research Ethics Board (#115810) and all participants provided informed implied consent by clicking a button to complete the survey prior to participating.

**Table 1:** Demographic information of pregnant women.

Measure	
N	804
Age, years, Median [IQR]	30 [27–33]
Gestational age, weeks, Median [IQR]	25 [17–32]
Country, % (n)	
USA	31.7% (255)
UK	20.6% (166)
Canada	20.5% (165)
India	10.7% (86)
Other	16.4% (132)
Pandemic month, % (n)	
May	27.9% (224)
June	5.0% (40)
July	61.4% (494)
August	5.7% (46)
Hours of sleep/night, Median [IQR]	7 [6–8]
Ethnicity, % (n)	
Caucasian	64.9% (522)
Asian	18.4% (148)
Black	5.6% (45)
Hispanic	3.3% (27)
Other	7.7% (62)
Education level††, Median [IQR]	18 [18–21]
*Perceived Stress Scale*	
Total score, Median [IQR]	20 [14–24]

Clinical and demographic factors IQR, interquartile range; PSS, perceived stress scale;

† Other countries included South Africa, the Philippines, Brazil, Croatia, Italy, Fiji;

††Education level, 18: College education, 21: Graduate degree

### Materials

Self-report measures used to assess maternal demographic characteristics, stress, sleep, physical activity levels, and diet were collected and are summarized below.

1. Demographic Characteristics
  Participants reported on gender, age, location (city, country), gestational age, due date, pregnancy complications, ethnicity, education level (years), occupation, days spent in quarantine/self-isolation, ability to see their healthcare provider, diagnosed maternal health problems (diabetes, cardiovascular health, depression), weight (pre-pregnancy, pregnancy), and height.
2. Antenatal Stress
  Maternal stress was assessed using the Perceived Stress Scale (PSS), a 10-item questionnaire [22] that has been validated for use in pregnant women [23]. The PSS measures the degree to which situations are appraised as stressful, and relates to experiences over the past month. The participant ranks each question on a scale of 0–4, and overall scores are obtained by reversing responses to four positively stated items and then summing across all scale items.
3. Sleep
  Maternal sleep patterns were assessed using the Pittsburgh Sleep Quality Index (PSQI), a validated 10-item questionnaire designed to measure sleep quality [24] that has been validated in pregnant women [25]. Items include hours of sleep, medication use, waking during the night, and trouble falling asleep. Scores for seven domains are calculated from the participant responses, and a global PSQI is assigned based on the sum of the seven component scores. As a previous relationship has been established between hours of sleep and maternal distress [16], the present study used the total hours of sleep as our measure of sleep quality.
4. Physical Activity
  Physical activity levels were assessed using the Pregnancy Physical Activity Questionnaire, a validated 36-item questionnaire [26]. Items include time spent doing housework (e.g., preparing meals), time spent in sedentary activities (e.g., watching TV), methods of transportation, and exercise. All response items are on frequency-based six-point Likert scales (e.g., ranging from “None” to “3 or more hours per week”). The nine subscales of the PPAQ were then scored according to questionnaire responses for the following types of activity: sedentary; light; moderate; vigorous; household/caregiving; occupational; sports/exercise; transportation; inactivity.
5. Diet
  Maternal food and beverage intake were assessed using PrimeScreen, a validated 21-item dietary screening questionnaire [27]. This questionnaire is a semiquantitative food frequency questionnaire allowing to assess diet quality. Items inquire about intake of various food groups including fruits and vegetables, dairy products, whole grains, fish, red meat, and major contributors to saturated and trans fats. The frequency of intake of foods is categorized by participants as: less than once per week (score = 1), once per week (score = 2), 2–3 times per week (score = 3), nearly daily or daily (score = 4), or twice or more per day (score = 5).

### Procedure

Data were collected online via Qualtrics, an online survey tool (Qualtrics, Provo, UT). After providing informed consent, participants completed all questionnaires. Completion time of the survey portion of the study was approximately 40 minutes.

### Data cleaning and reduction

Only data from the participants who completed all relevant questionnaire items were included in the analysis, and rows with missing values were removed, eliminating 69 participants. Rows with responses of ‘Prefer not to answer’ were also removed, eliminating 176 participants. After cleaning, a total of 804 datasets remained for analysis.

All code for feature selection and classification is available here: https://osf.io/6n2wk. Code for generating figures is available here: https://osf.io/z2raq. As there were a large number of questionnaire items, feature selection was performed in order to reduce complexity and improve the accuracy of the model [28]. Feature selection is a way of reducing the input features and strengthening the prediction results by including only relevant and meaningful features to the model [29]. Mutual information feature selection was used here to determine the optimal number of questionnaire items to maximize classification accuracy [28], and target classes for each model were determined in a hypothesis-driven manner, described below. All questionnaire responses were converted to numeric scales in order to perform feature selection.

**Fig 1:**
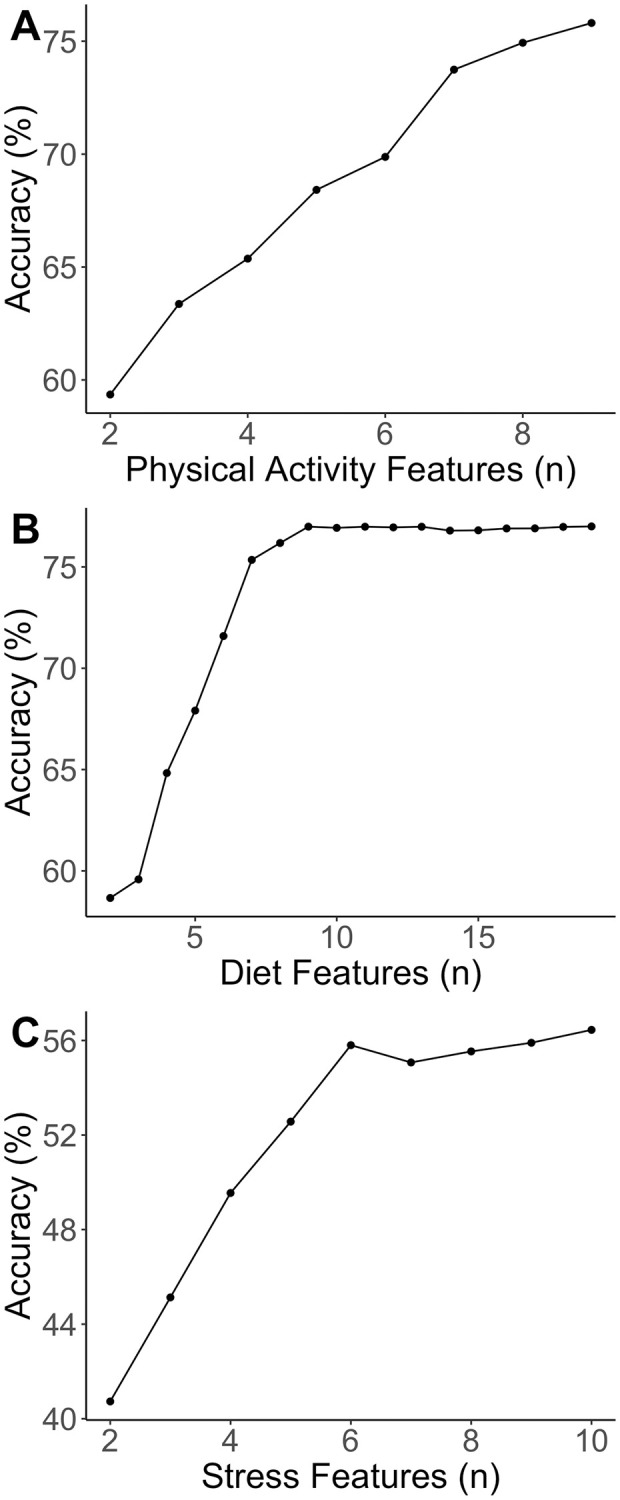
Accuracy values (%) during mutual information feature selection. A) physical activity predicting stress classes, B) diet predicting stress classes, C) stress predicting sleep classes.

For the first model, in which we examined whether physical activity features could accurately predict stress classes, nine physical activity-based questionnaire items were extracted for feature selection. Overall stress scores were divided into “low” and “high” target classes using a median split [30]. Each participant was then assigned to a target class of 0 (low stress levels) or 1 (high stress levels) based on their cluster membership. As can be seen in Fig 1A, classification accuracy was highest when all nine features of the PPAQ were included, and so no features were removed from further analyses [28].

In our second model, we examined whether dietary behaviours could accurately predict stress classes. We again divided overall stress scores into “low” and “high” target classes using a median split. Two questions pertaining to vitamin use were not used in the present study, resulting in 19 features being submitted to feature selection. As can be seen in Fig 1B, classification accuracy was highest when all 19 items of PrimeScreen were included.

For the third model in which we examined whether perceptions of stress could accurately classify sleep classes, feature selection again indicated that all 10 items of the PSS improved classification accuracy and were thus included in further analysis (Fig 1C). Participants’ sleep values were grouped into one of three classes based on the amount of sleep per night as follows: below 7 hours, 7–9 hours, and above 9 hours. These classes were based on prior work suggesting that pregnant women require between 7–9 hours of sleep per night [31].

### Support vector machine classification

Classification was performed using a multi-class Support Vector Machine (SVM) algorithm, a supervised learning model that finds a hyperplane in an N-dimensional space (with N being the number of features) in order to classify the observations. SVM parameters (i.e., kernel, C, and gamma) were first optimized using a grid search, which performs an exhaustive search over specified parameter values. Classifiers were then trained using the optimized set of parameters (Table 2) to identify classes from the set of features assigned for each prediction (Table 3). To overcome imbalance in class membership, we randomly sampled data points in each class based on the minority class. This ensures equal number of samples in each class. Data were then split into 80% train and 20% test sets, and trained and tested multi-class SVM on the data. Each iteration involved running the classifier on a random sampling of participants for the 80:20 train/test split. This procedure was repeated for 6000 iterations and the averaged prediction performance of the classifier over these iterations was reported. Averaging a large number of iterations ensured that no random sampling biased the classifier towards higher or lower performance. To perform above chance, a mean classification accuracy above 50% must be achieved for models with two target classes, and above 33% for models with three target classes. Baseline models were also generated to which we compared the trained models’ performance. The classification procedure was repeated 6000 times, however class labels were randomly shuffled for each iteration. Baseline classification levels are reported in Table 4. To determine whether classification accuracies were significantly above chance and baseline levels, one-sample, one-sided *t*-tests were computed with *μ* set to chance level.

**Table 2:** Default and optimized parameters for each predictive model.

Parameter	Default	Optimized		
		PPAQ → Stress	Diet → Stress	Stress → Sleep
Kernel	Radial Basis Function	Radial Basis Function	Radial Basis Function	Radial Basis Function
C	1	10	1	1
Gamma	Scale	1	1	1

**Table 3:** Number of features and target classes for each predictive model.

Cross predictor model	No. features	No. target classes
Physical activity → Stress	8	2
Diet → Stress	19	2
Stress → Sleep	10	3

**Table 4:** SVM mean classification accuracies and chance levels for three cross predictor models.

Cross predictor model	Accuracy	Chance level	Baseline Model
	M (SD)		
Physical activity → Stress	75.8% (3.7)	50%	50%
Diet → Stress	77.0% (3.2)	50%	50%
Stress → Sleep	56.4% (8.8)	33%	33%

### Feature ranking and target class characteristics

To determine which participant characteristics may explain target class membership, we examined the top three features that provided the most information gain. Proportions of total responses were calculated and plotted for each of the top three features, for each model, and Pearson’s chi-square tests were used to test the difference in distributions between classes.

## Results

### Support vector machine classification

**Fig 2:**
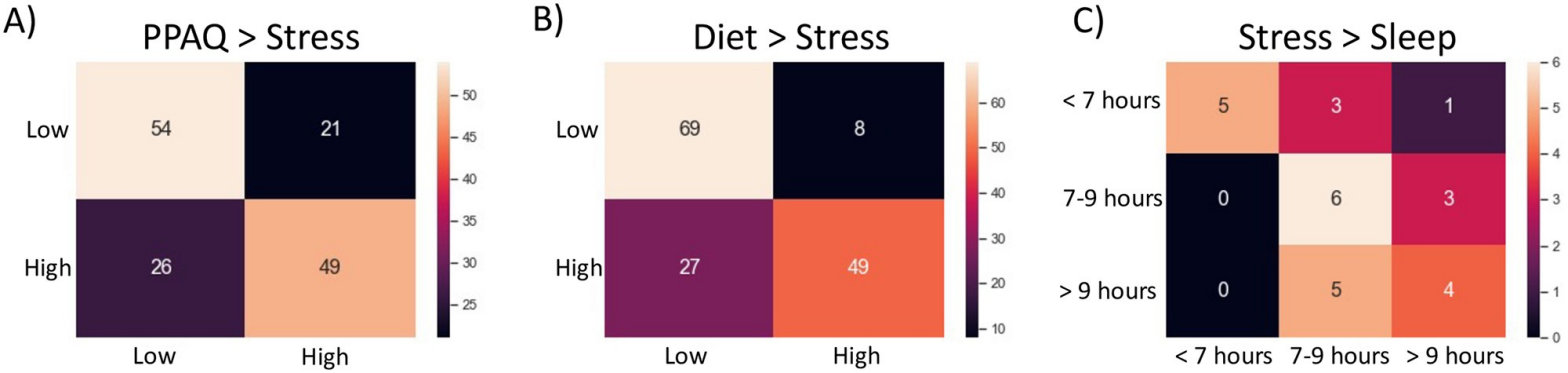
Confusion matrices of classifier performance. Classification models predicting A) stress class from physical activity; B) stress class from diet; and C) amount of sleep from perceived stress.

An SVM was used to determine whether, within pregnant women, physical activity could accurately predict perceived stress class. To test this, we averaged the SVM decoding accuracy across 6000 iterations; accuracy results are shown in Table 4 and precision and recall are shown in Table 5. Classification performance is shown in Fig 2. The SVM performed significantly above chance and baseline level (*t*(5999) = 541.21, *p* < .001), with a mean classification accuracy of 75.8% (SD = 3.7). In order to determine in turn whether dietary behaviours could accurately predict perceived stress class in pregnant women, a second SVM was run. The SVM again performed significantly above chance and baseline (*t*(5999) = 659.88, *p* < .001), with a mean classification accuracy of 77.0% (SD = 3.2). Finally, in order to determine in turn whether perceived stress could predict sleep class, a third SVM was run. The SVM again performed significantly above chance and baseline (*t*(5999) = 207.48, *p* < .001), with a mean classification accuracy of 56.4% (SD = 8.8).

**Table 5:** Evaluation of each model in terms of precision and recall.

Model	Class	Precision	Recall
PPAQ -> Stress	Low	0.77	0.75
	High	0.74	0.78
Diet -> Stress	Low	0.75	0.90
	High	0.93	0.64
Stress -> Sleep	< 7 hours	0.95	0.56
	7–9 hours	0.47	0.70
	> 10 hours	0.55	0.42

### Feature ranking and target class characteristics

**Fig 3:**
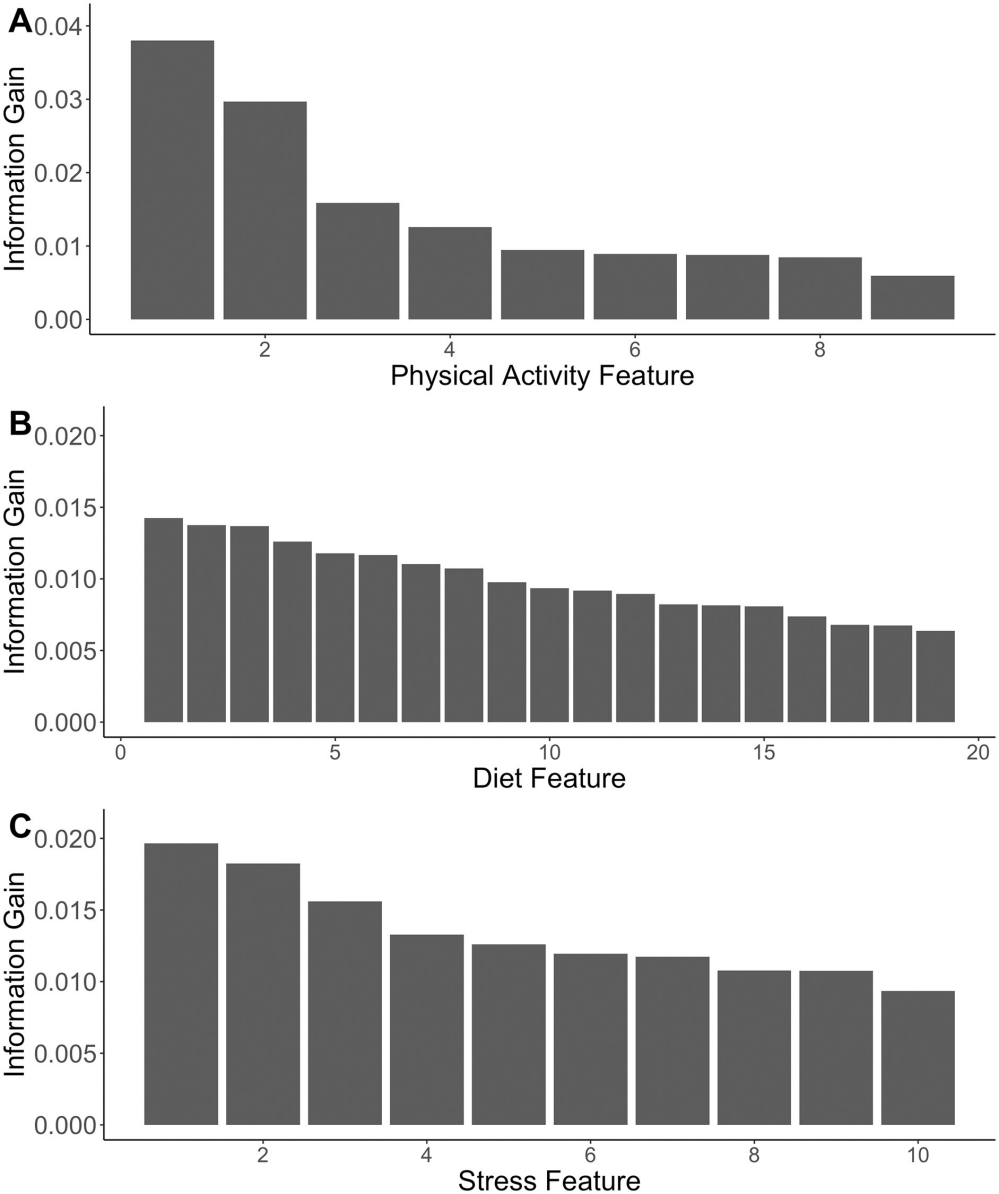
Features, ranked by amount of information gain they contribute. Classification models predicting A) stress class from physical activity; B) stress class from diet; and C) amount of sleep from perceived stress.

**Fig 4:**
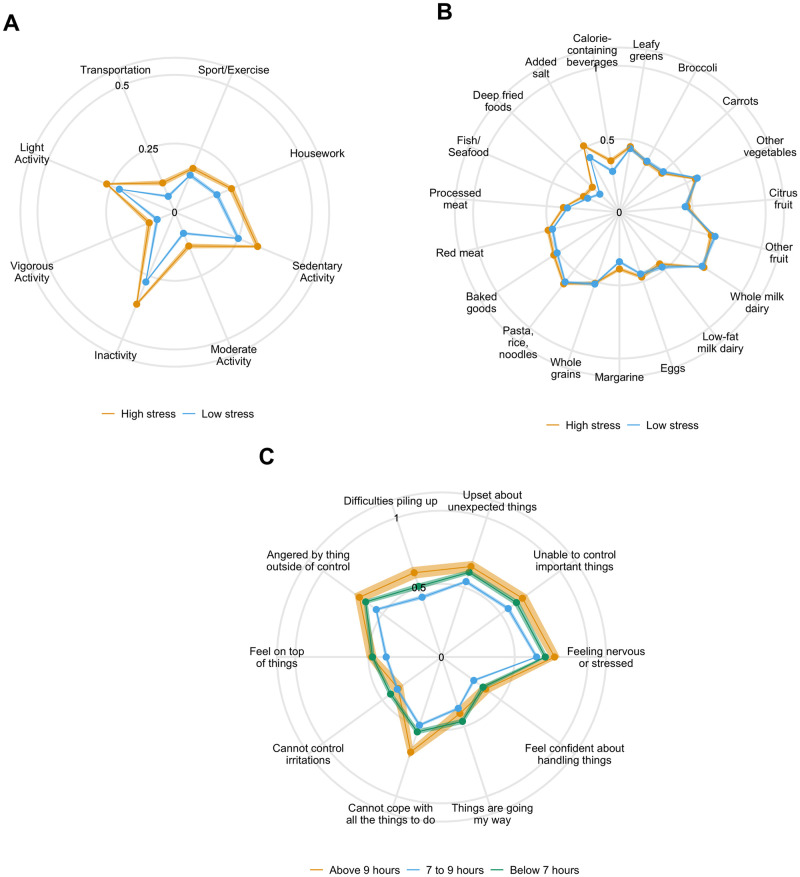
Distribution of mean responses by target class. A) Pregnancy Physical Activity Questionnaire features B) PrimeScreen features and C) Perceived Stress Scale features. Responses have been normalized to range from 0–1.

Feature ranking for each model is shown in Fig 3, and Table 6 shows the mapping of feature index to question. The top three features for each classifier were examined in order to describe the characteristics that contributed most to classification. In the case of physical activity, the three features that contributed the most information gain to stress classification were Sport/Exercise, Housework, and Sedentary Activity. Average responses for all features are shown in Fig 4A.

**Table 6:** Feature corresponding to feature index in Fig 3.

Feature index	Feature
Physical Activity	
1	Sports/Exercise
2	Household/Caregiving activity
3	Sedentary activity
4	Moderate activity
5	Inactivity activity
6	Vigorous activity
7	Light activity
8	Transportation
9	Occupational activity
Diet	*How often do you eat/drink…*
1	Add salt to food at the table
2	Deep fried foods (deep fried chicken, fish or seafood; French fries, onion rings)
3	Beef, pork or lamb as main dish
4	Pasta, rice, noodles
5	Fish/Seafood (not fried, but broiled, baked, poached, canned)
6	Calorie-containing beverages (e.g. regular soda, fruit drinks, Nestea, Gatorade)
7	Baked products (donuts, cookies, muffins, crackers, cakes, sweet rolls, pastries)
8	Margarine (stick-type not tub)
9	Carrots
10	Broccoli, broccoli rabe, cauliflower, cabbage, brussels sprouts
11	Whole milk dairy foods (whole milk, hard cheese, butter, ice cream)
12	Processed meats (sausages, salami, bologna, hot dogs, bacon)
13	Whole eggs
14	Dark green leafy vegetables (spinach, romaine lettuce, mesclun mix, kale, turnip greens, bok choy, swiss chard
15	Other fruits (e.g. fresh apples or pears, bananas, berries, grapes, melons)
16	Whole grain foods (e.g. whole grain breads, brown rice)
17	Citrus fruits (e.g. orange or grapefruit juice, oranges, grapefruits)
18	Low-fat milk products (e.g. low- fat/skim milk, yogurt, cottage cheese)
19	Other vegetables (e.g. peas, corn, green beans, tomatoes, squash)
Stress	*In the last month*, *how often have you felt or thought that…*
1	Things are going my way
2	I am on top of things
3	Angered by things out of my control
4	Difficulties piling up
5	Confident I can handle personal problems
6	I cannot cope with everything I have to do
7	I can control irritations in my life
8	I am upset by things happening unexpectedly
9	I cannot control the important things in my life
10	I feel nervous and stressed

The top three dietary features that contributed the most information gain to stress classification were “How often do you add salt to food at the table?”; “How often do you eat deep fried foods (deep fried chicken, fish or seafood; French fries, onion rings)?”; and “How often do you eat beef, pork or lamb as main dish?”. Average responses for each feature are shown in Fig 4B.

Finally, the top three stress features that contributed the most information gain to sleep classification were “In the last month, how often have you felt that things were going your way?”; “In the last month, how often have you felt that you were on top of things?”; and “In the last month, how often have you been angered because of things that were outside of your control?”. Average responses for each feature are shown in Fig 4C.

## Discussion

In the current study, we sought to determine whether physical activity and diet were resilience factors against prenatal stress during the pandemic, and whether perceived stress would in turn be predictive of hours of sleep, using an SVM trained on data from 804 pregnant women. Physical activity features were able to predict stress class (low/high) in pregnant women well above chance level; similarly, dietary behaviours were also able to predict stress class. When examining whether perceived stress could in turn predict sleep class in pregnant women, the classifier again performed significantly above chance level. These results suggest that physical activity and diet may be modifiable factors that could help to alleviate prenatal stress and promote maternal sleep. What choices, then, could pregnant women make in order to reduce stress levels and improve sleep?

To address this question, we examined which features provided the most explanatory power to the classifier. The top three physical activity features perhaps unsurprisingly included measures of intentional exercise such as walking, jogging, or swimming; housework; and sedentary activity. As shown in Fig 4A, while women with high stress levels reported marginally higher levels of exercise, they also reported higher levels of sedentary activity and housework. In fact, across all features, high stress women reported higher levels of physical activity.

The average responses to these physical activity features suggest that while research has shown that physical activity may be protective against stress, high amounts may be detrimental. It must also be noted that being required to spend large amounts of time doing these activities could itself be related to increased stress. For example, spending some time every day doing housework and active transportation is likely to have benefits, especially over more sedentary activities such as watching television. However, spending a lot of time doing these activities may be understandably stressful, or may represent a more stressful home environment, and thus may counteract the benefits of the physical activity. It has previously been reported that pregnant women who engaged in 150 minutes of physical activity per week showed better mental health outcomes than those who performed less [3]. Importantly, in the present study increased stress levels were associated with *higher* reported frequency of these activities in pregnant women, suggesting that too much physical activity may be just as detrimental to mental health as too little.

Dietary behaviours that proved to be most predictive of stress levels were related to adding salt at the table, eating fried foods, and eating red meat. Women who fell into the high stress class responded to these items as performing them more frequently than women who fell into the low stress class. In contrast to high stress women, the majority of women in the low stress class reported performing all three of these dietary behaviours less than once a week. Taken together, healthier dietary behaviours were predictive of lower stress levels, suggesting that this may be a modifiable lifestyle factor for promoting resilience to stress during pregnancy.

The stress features that most contributed to the prediction of sleep class also revealed an interesting pattern. Although pregnant women who were achieving too little or too much sleep appeared to feel more ‘on top of things’ and that ‘things were going their way’, they more frequently reported feeling unable to control their anger because of things outside of their control than women who achieved 7–9 hours of sleep per night. This form of stress is particularly present during the pandemic, where sudden, consequential changes to circumstances have been frequent. The direct relationship between this form of stress and sleep levels suggests that we may see increased levels of poor maternal outcomes during this period.

Although we reported the top three features for each classifier, it is important to remember that in all three models, many features contributed to the SVM’s performance, indicating that it is a *set* of choices surrounding each resilience factor that predicted stress class and sleep. The lifestyle factors we examined here are extremely multi-faceted, and ML techniques are able to account for complex relationships between variables [32]. This is promising for those pregnant women who may be finding it difficult to commit to moderate physical activity every day or to limiting unhealthy foods in their diet. That is, small changes to multiple habits, for example moderately reducing salt, fried foods, and fish, as well as other unhealthy foods, may have a combined beneficial effect and are easier to accomplish than cutting them out completely.

The present results provide important insight into the relationship between diet, physical activity, stress, and sleep in pregnant women; however, several limitations must be noted. Although the sample was large and heterogeneous in terms of age and location, sampling was limited to those pregnant women who had access to an internet-connected device and saw our study advertisement, possibly biasing the sample. Additionally, because the study was conducted online, it was not possible to confirm that participants were in fact pregnant. However, because compensation was minimal, the number of pregnancy-specific questions was large, and time to complete the study was approximately one hour, it is unlikely that non-pregnant individuals completed the entire study and therefore were included in the final analysis. Finally, the cross-sectional design limits conclusions that can be drawn about the cause and effect of our results; future work should aim to determine the causal relationship between these factors in pregnant women.

Psychological distress in pregnancy is a risk factor for adverse outcomes for maternal and neonatal health. It is critical to identify the signs, symptoms, and diagnostic thresholds that warrant prenatal intervention. This research adds to a developing consensus regarding adverse health behaviours concerning physical activity and diet, and the association with prenatal stress and the subsequent associations with sleep in pregnant women during the pandemic. Predictive modeling developed using ML-approaches may be used as a screening tool and to promote positive health behaviours for pregnant women who experience high levels of antenatal stress.
